# Protein *S*-palmitoylation: Potential strategy for inflammation-related diseases

**DOI:** 10.1016/j.jpha.2025.101490

**Published:** 2025-11-06

**Authors:** Sunan Li, Xinyue Dou, Jiamei Sun, Yongyuan Xiao, Tianyang Wang, Qiyuan Shan, Xin Han, Gang Cao

**Affiliations:** a School of Pharmacy, Zhejiang Chinese Medical University, Hangzhou, 310000, China; b The First Affiliated Hospital of Zhejiang Chinese Medical University, Hangzhou, 310000, China

**Keywords:** *S-*palmitoylation, Immune cell, Inflammation-related diseases, Pattern recognition receptors

## Abstract

*S*-palmitoylation represents a dynamic and reversible modification wherein palmitate forms covalent bonds with cysteine residues in proteins, thereby modifying their localization, stability, signaling pathways, and protein-protein interactions. Aberrations in *S*-palmitoylation are associated with the progression of various diseases, particularly inflammatory conditions. This association can be attributed to its regulation of pattern recognition receptors (PRRs) and its influence on immune cell functions, including macrophages, T cells, neutrophils, and natural killer cells (NK cells). This review synthesizes current knowledge regarding the impact of *S-*palmitoylation on inflammation-related diseases through examination of its regulatory roles in PRRs and immune cell functions. The objective is to illuminate the underlying mechanisms and regulatory networks involved in inflammation-related diseases.

## Highlights

• Palmitoylation covalently attaches palmitic acid to cysteine residues via a thioester bond, modulating protein-protein interactions.
• Palmitoylation modulates pattern-recognition receptors functions to influence inflammatory signal initiation, suggesting anti-inflammatory potential.
• Palmitoylation regulates immune cell function by targeting specific proteins, there by influencing the anti-inflammatory response.
• Regulating palmitoylation modification provides a potential target for treating various inflammatory diseases.

## Graphical abstract

**Figure:**
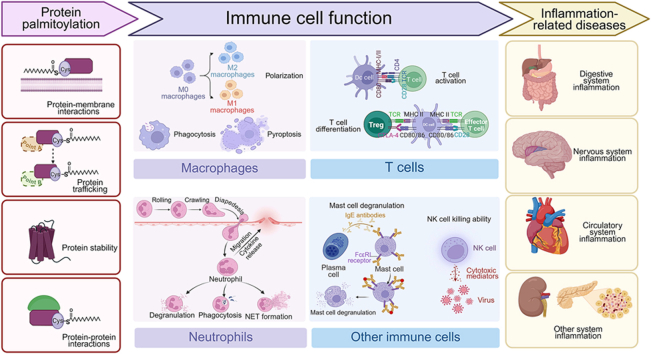

## 1 Introduction

**Fig. 1:**
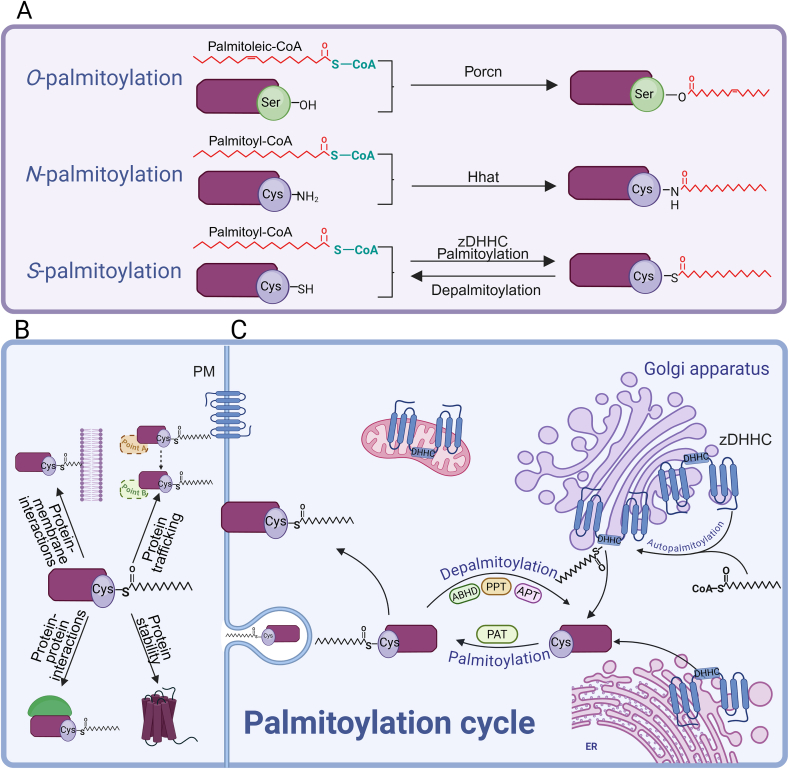
Protein *S-*palmitoylation and depalmitoylation. (A) Palmitoylation is classified into *S-*palmitoylation, *N*-palmitoylation, and *O*-palmitoylation according to the connection mode and modification sites. (B) *S-*palmitoylation affects protein membrane localization, subcellular trafficking, stability, and protein–protein interactions. (C) *S-*palmitoylation is linked to the cysteine residues of proteins through labile thioester bonds, and the dynamic equilibrium of the protein palmitoylation state can be achieved under the catalysis of palmitoyltransferases (PATs) and acyl-protein thioesterases (APTs). Zinc finger Asp-His-His-Cys motif-containing (zDHHC) family proteins that promote *S-*palmitoylation contain an Asp-His*-*His*-*Cys (DHHC) domain in which the cysteine residues are linked to palmitoyl-CoA through a thioester bond to form an acyl-enzyme intermediate. The palmitoyl group linked to the intermediate is then transferred to the substrate cysteine, completing the palmitoylation of the substrate protein. Porcn: porcupine *O*-acyltransferase; Hhat: hedgehog acyltransferase; PPT: palmitoyl-protein thioesterase; ABHD: α/β-hydrolase domain-containing protein; ER: endoplasmic reticulum.

Palmitoylation, a post-translational modification of proteins, can be categorized as *S-*palmitoylation, *N*-palmitoylation, or *O*-palmitoylation based on the linkage method and modification sites [1]. *N*-palmitoylation and *O*-palmitoylation involve attachments to glycine or serine residues of proteins through stable amide bonds and ester bonds, respectively [2,3], while *S-*palmitoylation connects to protein cysteine residues via labile thioester linkages [4] (Fig. 1A). *S-*palmitoylation is a reversible and dynamic post-translational modification of proteins [5]. This reversible lipid modification enhances protein membrane affinity, subcellular localization, stability, and protein-protein interactions [[6], [7], [8]], thereby contributing to the biological functions of multiple cell types. Significantly, *S-*palmitoylation constitutes a subset of *S-*acylation, which encompasses modifications where any long-chain fatty acid (including palmitic acid, stearic acid, oleic acid, etc.) links to protein cysteine via thioester bonds [9], whereas *S-*palmitoylation specifically indicates the modification where palmitic acid conjugates to cysteine residues through thioester bonds [8]. Among various types of *S-*acylation, *S-*palmitoylation represents the most prevalent form and demonstrates extensive involvement in the treatment of various diseases [[10], [11], [12], [13], [14]]. Recent studies indicate that *S-*palmitoylation modification plays a crucial role in regulating immune cell functions [15]. This modification process significantly influences the initiation and progression of inflammatory responses by activating innate and adaptive immune signaling pathways, thus participating in diverse pathophysiological processes [6]. Given the critical role of *S-*palmitoylation modification in various human diseases, Blanc et al. [16] established the corresponding “SwissPalm” database. Therefore, this study primarily focuses on examining *S-*palmitoylation and its mediated biological functions.

Inflammatory diseases, including rheumatoid arthritis, atherosclerosis, and sepsis, arise from deregulated immune activation and sustained inflammatory responses. Pattern recognition receptors (PRRs), key mediators in inflammatory processes, function as molecular sensors for pathogen-associated molecular patterns (PAMPs) and damage-associated molecular patterns (DAMPs) [17,18]. Upon activation, PRRs initiate downstream signaling cascades, such as the nuclear factor kappaB (NF-κB) and mitogen-activated protein kinase (MAPK) pathways [19,20], which orchestrate the inflammatory responses of immune cells, including macrophages, T cells, and neutrophils. These cells serve dual roles in both host defense and tissue repair, though their dysregulation may lead to chronic inflammation and tissue damage. Considering the vital role of PRRs in immune activation, understanding how *S-*palmitoylation modulates PRR signaling and immune cell function could establish a foundation for developing precision therapies targeting protein modifications in inflammatory diseases.

In this review, we comprehensively examine the molecular mechanisms and functional implications of *S-*palmitoylation, particularly focusing on its regulatory roles in PRR signaling and immune cell biology. The discussion concludes with an analysis of the pathophysiological roles of *S-*palmitoylation in various inflammatory diseases, aiming to establish a framework for translating these insights into innovative therapeutic strategies.

## 2 Protein *S-*palmitoylation and depalmitoylation: dynamic and reversible cycling

The chemical essence of *S-*palmitoylation involves palmitic acid covalently binding to protein cysteine residues [8], subsequently influencing protein functions through regulation of membrane localization, subcellular trafficking, stability enhancement, protein-protein interactions, and signal transduction (Fig. 1B). Initially, *S-*palmitoylation increases protein hydrophobicity through palmitoyl group conjugation, facilitating membrane binding and influencing protein localization [21]. Additionally, *S-*palmitoylation facilitates protein anchoring in lipid rafts, specialized regions of the cell membrane where critical signaling processes occur [22,23]. This lipid raft localization enables protein participation in signal transduction. Furthermore, palmitic acid group addition can alter protein conformation, reducing proteasomal recognition and degradation, thereby enhancing protein stability [24]. Lastly, *S-*palmitoylation modifies the three-dimensional (3D) structure of proteins, affecting their activity and molecular interactions [25,26].

Due to the instability of thioester bonds, *S-*palmitoylation undergoes regulation through the catalysis of palmitoyltransferases (PATs) and depalmitoylases, achieving a dynamic equilibrium of protein *S-*palmitoylation states [27] (Fig. 1C). PATs that catalyze protein *S-*palmitoylation reactions are commonly known as zinc finger Asp-His-His*-*Cys motif-containing (zDHHC) enzymes, as they contain an Asp-His-His*-*Cys domain [28]. In this domain, the cysteine residue forms a thioester bond with palmitoyl-CoA, creating an acyl-enzyme intermediate. Subsequently, the palmitoyl group transfers from the intermediate to the substrate cysteine, completing the protein *S-*palmitoylation [29,30]. Research has identified 23 zDHHC enzymes in humans [31], most of which are located in the Golgi, endoplasmic reticulum (ER), and plasma membrane [32,33]. Beyond the well-characterized localization to the ER, Golgi, and plasma membrane, several zDHHCs are targeted to other organelles, including mitochondria (e.g., zDHHC3) [34], endosomes/lysosomes (e.g., zDHHC2/20) [33,35], and vesicle (e.g., zDHHC23) [33]. In brief, zDHHC3 has been reported to promote the ubiquitin-mediated degradation of mitochondrial antiviral signaling protein (MAVS), thereby playing a critical role in antiviral innate immunity [34]. Additionally, zDHHC20 colocalizes with interferon-induced transmembrane protein 3 (IFITM3) at lysosomes, and modulates the activity of IFITM3 by enhancing its palmitoylation [35]. These enzymes collaborate across different organelles to complete the *S-*palmitoylation of numerous proteins, thereby influencing protein activity, subcellular transport, and signal transduction, ultimately enabling precise regulation of cellular function.

Furthermore, *S-*palmitoylation undergoes regulation by depalmitoylases due to its dynamic and reversible characteristics [36]. The main depalmitoylases include the acyl-protein thioesterase (APT) family, the palmitoyl-protein thioesterase (PPT) family, and the α/β-hydrolase domain-containing protein (ABHD) family [1]. The PPT family comprises PPT1 and PPT2, which operate in lysosomes and participate in protein depalmitoylation [37]. APT1 and APT2, serving as core depalmitoylases, dynamically modify essential signaling proteins, including MAVS, glutathione peroxidase 4 (GPX4), signal transducer and activator of transcription 3 (STAT3), and beta-catenin (β-catenin), thereby regulating immune responses [38], cell death [39], metabolic homeostasis [40,41], and renal fibrosis progression [42]. The ABHD family includes ABHD17 A/B/C and ABHD10. ABHD17A/B/C regulates the palmitoylation of substrate proteins such as postsynaptic density protein 95 (PSD95) and neuroblastoma RAS viral oncogene homolog (N-Ras), affecting neurodegenerative disease progression and tumor development [43,44]. ABHD10 mediates peroxiredoxin 5 (PRDX5) depalmitoylation, thereby influencing reduction-oxidation (REDOX) homeostasis [45]. Recent research reveals that ABHD8 recruits PAT zDHHC12 to mediate nucleotide oligomerization domain (NOD)-like receptor thermal protein domain-associated protein 3 (NLRP3) palmitoylation, promoting its degradation through autophagy [46], indicating that beyond auto-depalmitoylation, the ABHD family affects protein function through PAT recruitment.

In summary, PATs and depalmitoylases regulate protein *S-*palmitoylation dynamically and reversibly based on cellular physiological requirements, facilitating protein translocation between subcellular compartments and modulating their functional activities under varying physiological conditions.

## 3 Role of *S-*palmitoylation in PRRs

Inflammation initiation typically involves PAMPs, DAMPs, and PRRs [17,18]. *S-*palmitoylation plays a crucial role in the complex relationship between protein stability and signal transduction. Understanding *S-*palmitoylation's role in inflammation requires evaluation of its relationship with PRRs. Currently, PRRs primarily encompass toll-like receptors (TLRs), NOD-like receptors (NLRs), retinoic acid-inducible gene I-like receptors (RLRs), C-type lectin-like receptors (CLRs), and intracellular DNA sensors [47]. These receptors participate in the development of inflammation by recognizing various ligands, including PAMPs and DAMPs. *S-*palmitoylation influences different PRR functions, enabling precise intervention in inflammatory diseases. Given limited research on CLR *S-*palmitoylation, this discussion primarily focuses on *S-*palmitoylation's role in TLRs, NLRs, RLRs, and intracellular DNA sensors.

**Fig. 2:**
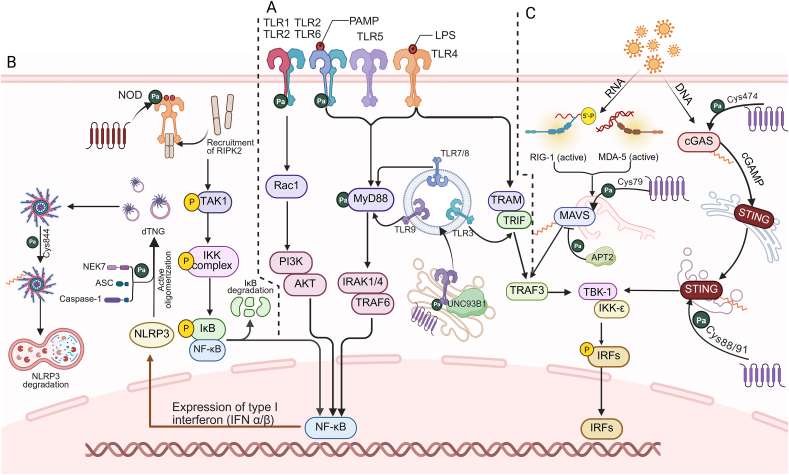
Palmitoylation of pattern recognition receptors (PRRs). (A) The toll-like receptors (TLRs) on the cell membrane and in endosomes can be palmitoylated. The palmitoylation of TLR2 and its downstream adaptor protein myeloid differentiation primary response protein 88 (MyD88) promotes their interaction. The palmitoylation-depalmitoylation cycle of endosomal TLR9 facilitates its transport and release from the Golgi to endosomes. (B) The palmitoylation of nucleotide oligomerization domain-containing protein 1/2 (NOD1/2) receptors in the cytoplasm leads to their localization on the intracellular membrane. Palmitoylation at different cysteine sites of NOD-like receptor thermal protein domain-associated protein 3 (NLRP3) impacts the activation, assembly, and degradation of the NLRP3 inflammasome, thus affecting inflammasome signal transduction. (C) Retinoic acid-inducible gene-I-like receptors (RLRs) and intracellular DNA sensors that recognize intracellular viral RNA and DNA, respectively, undergo palmitoylation. The palmitoylation of the RLR-downstream adaptor protein mitochondrial antiviral-signaling protein (MAVS) promotes its aggregation and localization in the mitochondria and its interaction with melanoma differentiation-associated protein 5 (MDA5) and retinoic acid-inducible gene I (RIG-I). The palmitoylation of cyclic guanosine monophosphate-adenosine monophosphate synthase (GMP-AMP) synthase (cGAS) and stimulator of interferon genes (STING), two intracellular DNA sensors, affects their interaction with double-stranded DNA (dsRNA) and their aggregation in the *trans-*Golgi network (TGN). P: phosphorylation; Pa: *S-*palmitoylation; TAK1: transforming growth factor-β-activated kinase 1; IKK: IκB kinase; NEK7: never in mitosis gene a (NIMA)-related kinase 7; Caspase-1: cysteine-dependent aspartate-specific protease-1; Rac1: ras-related C3 botulinum toxin substrate 1; PI3K: phosphoinositide 3-kinase; AKT: protein kinase B; TRIF: toll/interleukin-1 receptor (TIR)-domain-containing adaptor inducing interferon-β; TRAM: TRIF-related adaptor molecule; TRAF3: tumor necrosis factor (TNF) receptor-associated factor 3; APT2: acyl-protein thioesterase 2; cGAMP: cyclic guanosine monophosphate-adenosine monophosphate; TBK-1: TRAF family member-associated NF-κB activator (TANK)-binding kinase 1; IRF: interferon regulatory factor.

The TLR family can be categorized into TLR1/2/4/5 situated on the cell membrane and TLR3/7/8/9 positioned on the endosomal membrane based on their cellular localization. *S-*palmitoylation commonly occurs on TLR2 and TLR9, likely due to their functional requirements. For TLR2, *S-*palmitoylation primarily occurs at Cys609 through the action of zDHHC2/3/6/7/15, activating the downstream NF-κB signaling pathway [48]. For TLR9, Cys258 and Cys265 undergo *S-*palmitoylation induced by zDHHC3, enabling TLR9's movement from the Golgi to the endosome. The depalmitoylation mediated by PPT1 within the endosome facilitates the release of TLR9 from Unc-93 homolog B1 (UNC93B1). This *S-*palmitoylation and depalmitoylation cycle regulates TLR9's binding to ligands and the production of interferon (IFN) and tumor necrosis factor (TNF) cytokines, key mediators in inflammation [49] (Fig. 2). Additionally, the *S-*palmitoylation of TLR adaptors myeloid differentiation primary response 88 (Myd88) at Cys113 and Cys274 strengthens their interaction with TLRs [50], effectively activating the downstream NF-κB and MAPK signaling pathways that induce inflammatory factor expression.

In contrast to TLRs, NLRs are intracellular receptors that primarily recognize pathogens and damage signals within the cell. NOD1/2 and NLRP3, the most extensively studied NLRs, depend on *S-*palmitoylation for their stability and assembly. For NOD1/2, the Cys558/567/952 sites are palmitoylated under zDHHC5 catalysis, enabling their transfer to the cell intima and recruitment of receptor-interacting protein 2 (RIP2) to activate NF-κB and MAPK signaling pathways [51]. The *S-*palmitoylation of NLRP3 at Cys130/901 directs its localization to the *trans*-Golgi network (TGN), promoting NLRP3 inflammasome activation [[52], [53], [54]]. zDHHC5 and zDHHC7 palmitoylate the Cys837/838/419 sites, strengthening the interaction between NLRP3 and never in mitosis gene a (NIMA)-related kinase 7 (NEK7) and facilitating NLRP3 inflammasome assembly [55]. zDHHC12 induces *S-*palmitoylation at Cys844, promoting NLRP3's autophagic degradation and halting inflammasome signaling [56] (Fig. 2). This evidence demonstrates that *S-*palmitoylation is essential for controlling NOD1/2 and NLRP3 inflammasome activity.

RLRs are predominantly located in the cytoplasm and are mainly classified into retinoic acid-inducible gene I (RIG-I) and melanoma-differentiation-associated gene 5 (MDA5). Unlike NLRs that recognize intracellular PAMPs and DAMPs, RLRs specifically recognize intracellular viral double-stranded RNA (dsRNA) and subsequently activate downstream signaling pathways and induce type I IFNs and inflammatory cytokines expression. As a downstream adaptor protein of RLRs, MAVS undergoes *S-*palmitoylation at Cys79 through zDHHC24 and zDHHC12, enhancing MAVS stabilization and activation [38,57]. This process facilitates MAVS aggregation and localization in the mitochondria, thereby strengthening MAVS interactions with MDA5, RIG-I, and TNF receptor-associated factor (TRAF) family member-associated NF-κB activator (TANK) binding kinase 1 (TBK1), amplifying the downstream IFN response [58], and ultimately serving a crucial role in antiviral immunity (Fig. 2).

Intracellular DNA sensors constitute a family of PRRs that identify DNA fragments within cells. *S-*palmitoylation modifies two significant intracellular DNA sensors, cyclic cyclic guanosine monophosphate-adenosine monophosphate synthase (GMP-AMP) synthetase (cGAS) and stimulator of interferon genes (STING). The *S-*palmitoylation of cGAS at Cys474 limits its interaction with dsDNA [59], affecting subsequent signal conduction. The STING located downstream of cGAS also undergoes *S-*palmitoylation. Its Cys88/91 sites are palmitoylated under zDHHC3/7/15, promoting STING aggregation in the TGN [60,61] (Fig. 2); recruiting downstream signal molecules; and activating TBK1, interferon regulatory factor 3 (IRF3), and NF-κB signal transduction, thus regulating immune response.

**Fig. 3:**
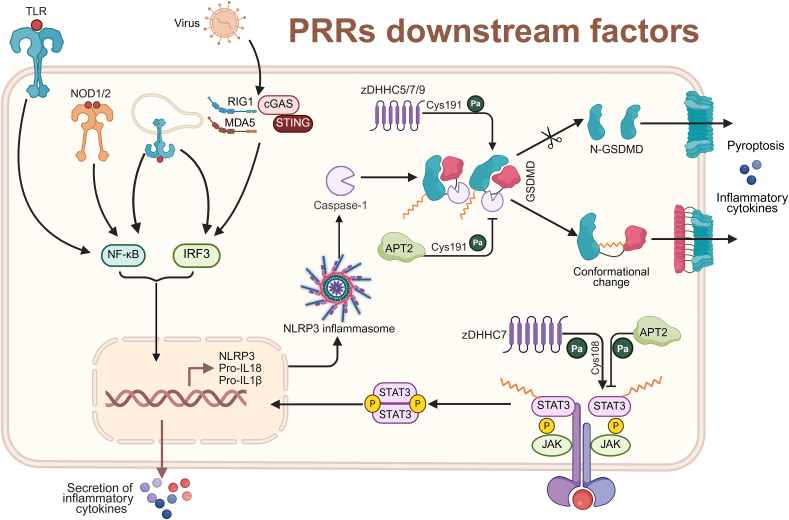
Palmitoylation of pattern recognition receptors (PRRs) downstream factors. The palmitoylation of the transcription factor signal transducer and activator of transcription 3 (STAT3) and gasdermin D (GSDMD) downstream of PRRs influences their binding to janus kinase (JAK) and the phosphorylation of STAT3 at the Y705 site, activates GSDMD, and alters the conformation of GSDMD and the occurrence of pyroptosis.

*S-*palmitoylation modulates the activities of PRRs and modifies the transcription factor STAT3 downstream of PRRs and gasdermin D (GSDMD), an executor of pyroptosis. Upon recognition of external stimuli by PRRs, STAT3 *S-*palmitoylation facilitates its translocation to the cell membrane, activating the janus kinase (JAK)-STAT3 signaling pathway [62], thereby regulating immune response and inflammatory processes [63]. Recent research has highlighted the significance of GSDMD *S-*palmitoylation. The *S-*palmitoylation at the Cys191 site of GSDMD enhances its translocation to the cell membrane and formation of plasma membrane pores [[64], [65], [66], [67]], potentially altering GSDMD conformation and facilitating pyroptosis [68,69] (Fig. 3).

In summary, *S-*palmitoylation modulates inflammatory signal transduction through dynamic regulation of PRRs' localization, stability, activity, and signaling complex formation, thereby affecting inflammatory cytokine transcription and release, underscoring its central role in inflammatory responses. Consequently, the impact of PRR *S-*palmitoylation on immune cell functions will be detailed in subsequent sections.

## 4 *S-*palmitoylation in immune cells

PRRs, essential components of the innate immune system, are primarily expressed on the surface or within the cytoplasm of immune cells. Following recognition of PAMPs and DAMPs, PRRs activate downstream signaling pathways in immune cells, which is essential for immune cell function during inflammation [19,20]. Understanding the regulatory role of *S-*palmitoylation in immune cell functionality holds significant implications for treating inflammation-related diseases.

### 4.1 Macrophages

**Fig. 4:**
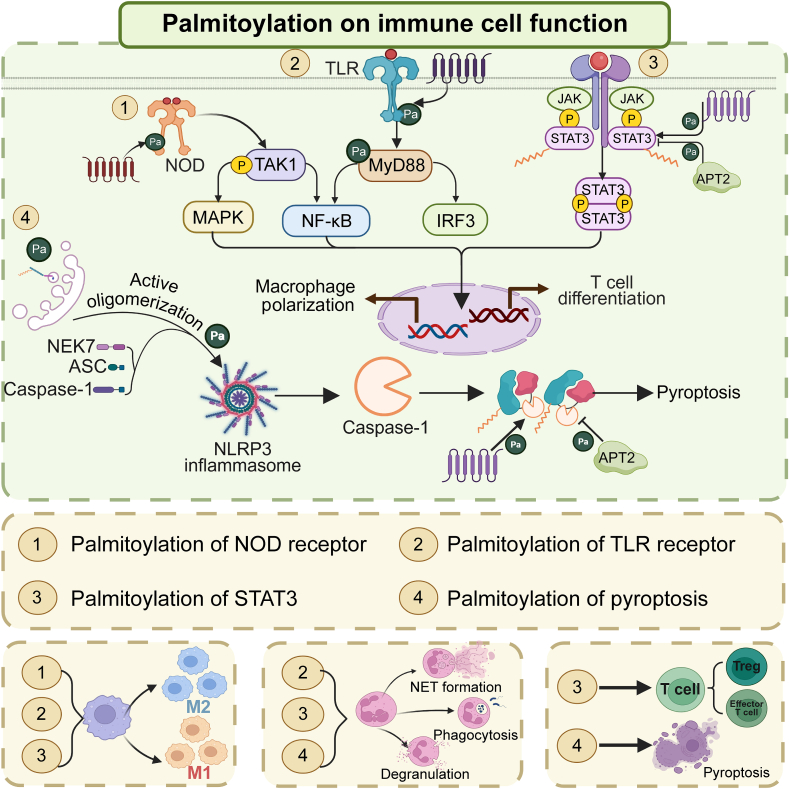
Regulation of immune cell function by palmitoylation. Palmitoylation regulates the polarization, pyroptosis, and phagocytic function of macrophages by affecting the palmitoylation of pattern recognition receptors (PRRs), the downstream signal transducer and activator of transcription 3 (STAT3) transcription factor, and pyroptosis, thus influencing the immune function of macrophages. Palmitoylation affects T cell differentiation by regulating STAT3 activities, thereby modulating the role of T cells in inflammation. It can influence the chemotaxis of neutrophils and the formation of neutrophil extracellular traps (NETs) through toll-like receptors (TLRs), pyroptosis, and STAT3, thereby improving the initiation and development of inflammatory disorders.

During inflammatory responses, macrophages differentiate into pro-inflammatory M1 type and anti-inflammatory M2 type under different stimuli. During M1 macrophage polarization, *S-*palmitoylation of PRRs, including NOD1/2 and TLR4, along with their adaptor protein Myd88, substantially enhances their membrane localization and signal transduction capabilities. Upon activation, NLRs and TLRs effectively recruit downstream signal molecules and activate signaling pathways such as NF-κB and MAPK [50,51], promoting M1 macrophage-related gene expression and enhancing macrophage pro-inflammatory activity. Sustained M1 macrophage activation leads to macrophage pyroptosis, tissue damage, and inflammatory disease progression [70]. STAT3, a critical molecule in cytokine signaling, plays an essential regulatory role in M2 macrophage polarization [71]. *S-*palmitoylation may enhance STAT3 nuclear translocation and DNA binding [72], regulating M2 macrophage-related gene transcription and expression, thus enabling macrophage functions including anti-inflammatory effects, tissue repair, and immunomodulation [73] (Fig. 4). These latter functions help maintain tissue homeostasis and repair damaged tissues.

**Fig. 5:**
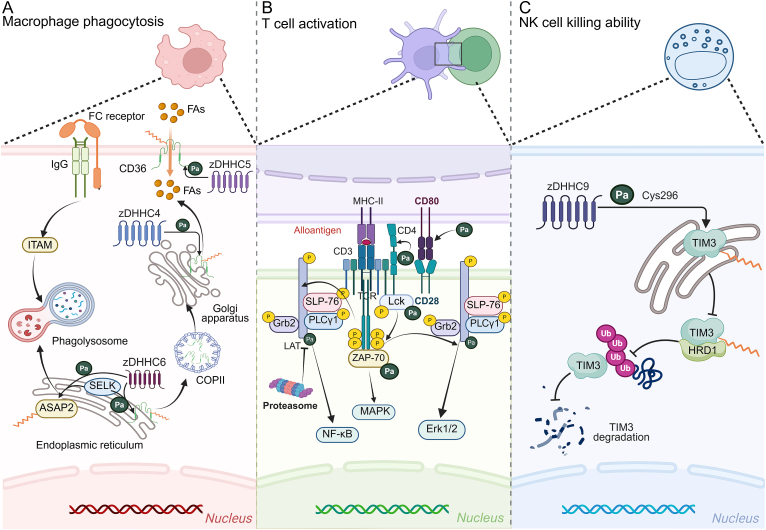
Palmitoylation targets other proteins to regulate the function of immune cells. (A) The palmitoylation of macrophage Selenoprotein K (SELK)-dependent proteins, ankyrin repeat and PH domain 2 (ASAP2), and cluster of differentiation 36 (CD36) indirectly affected the phagocytosis of macrophages. (B) The palmitoylation of T cell co-receptor CD4, co-stimulatory signal CD80, and its downstream lymphocyte-specific protein tyrosine kinase (LCK), zeta-chain-associated protein kinase of 70 kDa (ZAP-70), and linker for activation of T cells (LAT) were beneficial to T cell activation. (C) The palmitoylation of T-cell immunoglobulin and mucin-domain containing protein 3 (TIM-3) Cys296 on the surface of natural killer cells (NK cells) promotes the stability of TIM-3, thereby enhancing the killing ability of NK cells. ER: endoplasmic reticulum; FAs: fatty acid; ITAM: immunoreceptor tyrosine-based activation motif; MHC-II: major histocompatibility complex class II; SLP-76: src homology 2 domain-containing leukocyte protein of 76 kDa; PLCγ1: phospholipase C gamma 1; Grb2: growth factor receptor-bound protein 2; MAPK: mitogen-activated protein kinase; Erk1/2: extracellular signal-regulated kinase 1/2; HRD1: β-Hydroxy β-Methylglutaryl-CoA reductase degradation protein 1.

Macrophage inflammasome *S-*palmitoylation merits particular attention. *S-*palmitoylation at specific NLRP3 sites influences its activation, membrane localization, and apoptosis*-*associated speck-like protein containing a caspase recruitment domain (ASC) recruitment in macrophages [52,54,55]. Additionally, GSDMD *S-*palmitoylation promotes macrophage membrane pore development, a process inhibited by palmitoylase APT2, thereby reducing macrophage pyroptosis and downstream pro-inflammatory molecule production, including interleukin-18 (IL-18) and IL-1β [64,65,67] (Fig. 4). Macrophage surface Fc receptors and scavenger receptors, associated with phagocytic function, also undergo *S-*palmitoylation. In this process, Selenoprotein K (SELK)-dependent *S-*palmitoylation of ankyrin repeat and PH domain 2 (ASAP2) Cys86, induced by zDHHC6/SELK, enhances its membrane binding in the phagocytic cup and indirectly affects macrophage endocytosis [74] (Fig. 5). Similarly, SELK-dependent *S-*palmitoylation of protein-CD36 enhances its plasma membrane translocation under zDHHC4/5/6/SELK induction and maintains its plasma membrane localization and fatty acid absorption [75,76].

In summary, *S-*palmitoylation can influence the polarization of macrophages, potentially enhancing their phagocytic capacity and modulating the secretion of inflammatory factors, which are essential for the regulation of the body's inflammatory response, though the exact nature and extent of this regulation may depend on the specific cellular context and signaling pathways involved.

### 4.2 T cells

As a fundamental component in regulating the body's inflammatory response, T cells undergo rapid proliferation and differentiation into effector T cells upon recognition of foreign antigen stimulation, thereby executing their immunomodulatory function [77]. The initial activation of T cells requires specific recognition of homologous antigens presented by antigen-presenting cells (APCs) through the T cell receptor (TCR) on their surface and its coreceptors (such as cluster of differentiation 4/8 (CD4/8)) [78]. During this process, CD4 undergoes specific *S-*palmitoylation at two membrane-proximal cysteine residues, Cys396 and Cys399, enhancing its localization in lipid rafts [79]. Following CD4/8 binding to the nonpolymorphic regions of major histocompatibility complex class II (MHC II) molecules, substantial amounts of lymphocyte-specific protein tyrosine kinase (Lck) are recruited [79,80]. The *S-*palmitoylation of Cys3 and Cys5 in Lck facilitates its targeting to the plasma membrane, TCR signal transduction, and T cell activation [81]. This modification also promotes Lck interaction with the TCR-CD3 complex, thereby recruiting and inducing the activation of the ζ-chain-associated zeta chain of T cell receptor-associated protein kinase 70 (ZAP-70) [82,83]. Cys3 demonstrates greater efficacy than Cys5 in targeting plasma membrane localization and T cell activation [84]. The *S-*palmitoylation of ZAP-70 at Cys564 downstream of Lck extends the interaction between ZAP-70 and its activating kinase Lck, resulting in increased ZAP-70 phosphorylation [85]. Mutation of this site inhibits the phosphorylation of its downstream target adaptor proteins linker for activation of T cells (LAT) and SH2 domain-containing leukocyte phosphoprotein of 76 kDa (SLP-76) [81], disrupting the TCR signaling chain (Fig. 5). LAT *S-*palmitoylation at Cys26 and Cys29, crucial for T cell development and function, enables its targeting of glycolipid-rich microdomains and prevents its proteasomal degradation [86]. Inhibition of LAT *S-*palmitoylation affects its phosphorylation by upstream tyrosine kinases [87], thereby suppressing T cell signal transduction and activation processes. Additionally, T cell activation requires interaction between surface costimulatory molecules, such as CD28, and corresponding APC surface ligands, like B7 family molecules, for complete activation [78,88]. During this process, CD80, the CD28 ligand, undergoes *S-*palmitoylation at Cys261/262/266/271 in the transmembrane and cytoplasmic regions under zDHHC20 induction. This modification protects CD80 from ubiquitination-mediated degradation and ensures its precise plasma membrane localization [89], facilitating costimulatory signal release and T-cell activation.

STAT3, a principal transcription factor in Th17 cell differentiation [90], undergoes *S-*palmitoylation at Cys108 through zDHHC7. This modification induces its plasma membrane translocation and phosphorylation by JAK2. APT2 selectively depalmitoylates p-STAT3, promoting its nuclear translocation and subsequent activation [63]. This dynamic *S-*palmitoylation cycle drives STAT3 activation and enhances its binding to Th17-related gene promoter regions, thereby influencing Th17 cell differentiation [91,92]. Upon nuclear entry following depalmitoylation, p-STAT3 binds to the forkhead box protein P3 (Foxp3) promoter, a transcription factor associated with Treg cell differentiation [93,94] (Fig. 4). This binding suppresses Foxp3 transcriptional activity, affecting Treg cell differentiation and function. Foxp3 undergoes *S-*palmitoylation at Cys204, Cys218, Cys280, Cys281, and Cys424 sites through zDHHC2/3/7 induction. This modification influences Foxp3 nuclear localization and stability, consequently affecting Treg differentiation and function. Conversely, the absence of Foxp3 *S-*palmitoylation significantly decreases nuclear Foxp3 expression and inhibits T cell differentiation into Tregs [95].

In summary, *S-*palmitoylation serves a vital regulatory function in T cell activation and functional modulation, demonstrating significant importance in inflammatory disease pathogenesis and progression.

### 4.3 Neutrophils

Neutrophils constitute the predominant white blood cell population and rapidly migrate to inflammatory sites to execute anti-inflammatory functions during infection or tissue injury [96,97]. During this process, *S-*palmitoylation influences TLR signaling pathway activation and NLRP3 inflammasome function, resulting in inflammatory factor release and neutrophil migration to infection sites for anti-inflammatory effects [50]. Research has demonstrated that STAT3 transcriptional activation in neutrophils upregulates GSDMD [98], significantly affecting neutrophil extracellular traps (NETs) release [98,99] (Fig. 4). Given that *S-*palmitoylation critically influences STAT3 transcriptional activity and GSDMD expression, it likely serves an essential regulatory function in NETs release. Furthermore, STING *S-*palmitoylation affects its binding to syntaxin-binding protein 2 (STXBP2), enhances platelet activation and NETs formation, ultimately contributing to septic thrombi formation [100]. In conclusion, *S-*palmitoylation significantly influences inflammatory diseases through its effects on neutrophil chemotaxis and NETs formation.

### 4.4 Other immune cells

Macrophages, T cells, and neutrophils regulate immunity through distinct mechanisms. The natural killer cells (NK cells) directly identify and eliminate virus*-*infected cells, effectively limiting pathogen spread [101]. Mast cells release numerous cytokines through degranulation [102], subsequently activating NK cells and enhancing their antiviral activity. During this process, *S-*palmitoylation modifies the Cys296 site of T-cell immunoglobulin and mucin-domain containing protein 3 (TIM-3), an important immune checkpoint molecule on NK cells, inhibiting its binding to the E3 ubiquitin ligase hydroxymethylglutaryl-CoA reductase degradation protein 1 (HRD1), reducing TIM-3 degradation, and promoting TIM-3 stability and membrane expression, thereby enhancing NK cell cytotoxicity and pathogen elimination capacity [103] (Fig. 5). The *S-*palmitoylation of prohibitin 1 (PHB1) in mast cell granules facilitates its targeted translocation to plasma membrane lipid rafts, promoting mast cell degranulation and cytokine secretion [104]. These mechanisms influence NK cell cytotoxicity and immune cell recruitment. In conclusion, *S-*palmitoylation affects NK cell cytotoxicity. Mast cell degranulation and cytokine secretion participate in immune regulation, contributing to inflammatory disorder initiation and progression.

*S-*palmitoylation maintains precise regulation over inflammatory signaling pathways through modulating membrane localization, stability, and interactions of critical signaling molecules in immune cells. This regulation influences immune cell function, differentiation, and cytokine production, serving a dual function in inflammatory disease pathogenesis, enhancing pathogen clearance while mitigating excessive inflammatory damage from immune dysregulation. Consequently, therapeutic agents targeting palmitoylation-modifying enzymes or their substrate proteins present promising opportunities for precise immune cell regulation, potentially offering innovative approaches for treating inflammation-related disorders.

## 5 Targeting protein *S-*palmitoylation is a novel strategy to treat inflammation-related diseases

Given its involvement in PRRs and regulation of immune cell function, *S-*palmitoylation demonstrates significant therapeutic potential for inflammation-related diseases. A comprehensive analysis examined the regulatory mechanisms of *S-*palmitoylation across human systems to establish a novel theoretical framework for preventing and treating inflammation-related diseases.

### 5.1 Digestive system inflammation

Digestive system inflammatory diseases primarily encompass intestinal and liver inflammation. Clinical studies indicate significantly elevated expression levels of zDHHC7 and APT2 in patients with intestinal inflammation compared to healthy individuals, indicating *S-*palmitoylation's potential role in intestinal inflammation pathogenesis.

In a dextran sulfate sodium-induced mouse model of intestinal inflammation, zDHHC17-mediated *S-*palmitoylation of NLRP3 at Cys419 in macrophages activates the NLRP3 inflammasome. The *S-*palmitoylation inhibitor 2-bromopalmitate (2-BP) effectively inhibits this process, reducing colonic pathology and intestinal inflammation [105]. Furthermore, the *S-*palmitoylation cycle of STAT3, regulated by zDHHC7 and APT2, promotes Th17 differentiation, influencing intestinal inflammation [41,63,106]. Chronic intestinal inflammation may trigger liver inflammation through the gut–liver axis. *S-*palmitoylation, as a reversible lipid metabolic modification, primarily regulates liver lipid metabolic diseases. Non-alcoholic steatohepatitis (NASH) patients exhibit increased *S-*palmitoylation of transmembrane protein CD36 on liver cell membranes, enhancing CD36 membrane localization and free fatty acid uptake. This process inhibits fatty acid β-oxidation, contributing to intracellular lipid accumulation and NASH progression [107].

NASH patients demonstrate significantly elevated zDHHC3 protein levels. zDHHC3 enhances membrane localization of inactive rhomboid protein 2 (IRHOM2) and prevents its ubiquitination-mediated degradation through palmitoylation at Cys476 [108], thereby accelerating NASH development. These observations demonstrate that *S-*palmitoylation regulates various digestive system inflammatory diseases through multiple signaling pathways, offering extensive opportunities for developing novel diagnostic methods and therapeutic strategies.

### 5.2 Inflammation in the nervous system

Neuroinflammation constitutes a critical factor in the pathogenesis and progression of numerous neurological disorders. Dysregulation of *S-*palmitoylation has been implicated in the initiation of neuroinflammatory processes through the disruption of glial cell functions, thereby contributing to the development of various neurological conditions.

In Alzheimer's disease (AD) patients and mouse models, downregulation of SELK has been shown to inhibit the *S-*palmitoylation of CD36, compromising microglial migration and phagocytosis of Aβ, which exacerbates neuroinflammation [109]. Similarly, Yang et al. [110] demonstrated that zDHHC21-mediated *S-*palmitoylation at transient receptor potential vanilloid 2 (TRPV2) Cys277 diminishes Aβ phagocytosis by microglia, thereby accelerating AD progression. Comparable to its function in the digestive system, *S-*palmitoylation facilitates activation of the NLRP3 inflammasome, which subsequently induces microglial polarization toward a pro-inflammatory M1 phenotype [111], enhancing cytokine secretion and amplifying central nervous system inflammation. Furthermore, *S-*palmitoylation contributes to the aggregation of multiple aberrant proteins acting as DAMPs in the nervous system, which can be recognized by PRRs and affect glial cell function. In AD mouse models, reduced *S-*palmitylation of PSD95 exacerbates the neurological dysfunction caused by Aβ accumulation [112,113]. Regarding amyloid precursor protein (APP), a source of Aβ, the palmitylation of its Cys186 and Cys187 enhances BACE1's cleavage of APP, resulting in increased Aβ production [114]. Similarly, *S-*palmitylation modifies synaptic binding protein 11 (Syt11) Cys39 and Cys40, protecting them from endolysosomal degradation, thereby enhancing α-synuclein (α-syn) binding to the intracellular membrane and promoting pathological α-syn aggregation in Parkinson's disease (PD) [115]. The abnormal accumulation of Aβ and α-syn induced by *S-*palmitoylation triggers microglial activation and impairs microglial autophagy and phagocytosis [116,117], consequently initiating neuroinflammation.

In conclusion, *S-*palmitoylation dynamically modulates the function of essential neural proteins, profoundly influencing glial cell states and contributing to the pathogenesis of multiple neurological disorders. Consequently, elucidating the mechanistic roles of *S-*palmitoylation within the nervous system is essential for advancing our understanding of neurological disease pathophysiology and for the development of innovative therapeutic strategies.

### 5.3 Inflammation in the circulatory system

Inflammatory diseases in the circulatory system primarily encompass conditions affecting blood vessels and the heart, including atherosclerosis, sepsis, and myocardial infarction. During disease progression, *S-*palmitoylation exhibits potential anti-inflammatory effects mainly through modulating immune cell biological functions and pyroptosis. In atherosclerosis, decreased *S-*palmitoylation of p110α, a crucial subunit of phosphatidylinositol 3-kinase (PI3K), promotes p110α nuclear translocation in macrophages and inhibits macrophage differentiation into foam cells, thus ameliorating this condition [118]. Inhibition of STING *S-*palmitoylation reduces neutrophil granule secretion and NET formation, suppressing septic thrombus development [100]. Additionally, *S-*palmitoylation of NLRP3 inflammasome modulates the inflammatory response in sepsis animal models [119,120]. The *S-*palmitoylations of different GSDMD sites by zDHHC7, APT2, and zDHHC14 influence GSDMD activation, thereby increasing septic mice survival rates [67] and reducing cardiomyocyte pyroptosis in acute myocardial infarction mice [121].

*S-*palmitoylation in vascular endothelial cells (VECs) warrants attention. In atherosclerotic mice fed a high-fat diet, extensive lipid droplet accumulation disrupts VEC lipid homeostasis, leading to decreased *S-*palmitoylation of ciliary proteins [122]. This alteration ultimately results in vascular endothelial dysfunction and atherosclerosis. As a negative regulator of angiogenesis, reduced *S-*palmitoylation of vascular endothelial growth factor receptor (VEGFR) significantly decreases its stability, thereby promoting angiogenesis and myocardial tissue repair [123]. In conclusion, *S-*palmitoylation influences circulatory system inflammatory diseases through multiple pathways. Targeting *S-*palmitoylation presents a promising therapeutic approach for inflammatory disorders within the circulatory system.

### 5.4 Inflammation in other systems

*S-*palmitoylation plays a significant role in the inflammatory responses of the aforementioned body systems, with notable effects on the urinary and endocrine systems. The kidney, as the primary organ of the urinary system, performs essential functions. Research indicates that in chronic kidney disease patients and mouse renal tubular cell models, zDHHC9 exhibits significant downregulation, which decreases the *S-*palmitoylation level of β-catenin at Cys300. This reduction inhibits ubiquitination-mediated degradation, subsequently accelerating renal fibrosis progression [42]. Within the endocrine system, *S-*palmitoylation demonstrates particular relevance to metabolic disorders, notably diabetes. Diabetic patients' islets show elevated APT1 messenger RNA (mRNA) levels, despite significantly reduced enzyme activity. The *S-*palmitoylation of secretory carrier membrane protein 1 (Scamp1), an APT1 substrate protein in islets and transmembrane vesicle protein, increases at Cys132, impacting insulin secretion [124]. These findings suggest that *S-*palmitoylation represents a potential therapeutic target for inflammatory diseases across multiple body systems.

In conclusion, *S-*palmitoylation extensively influences inflammation-related diseases throughout multiple systemic systems by regulating protein membrane localization, stability, and interactions. Future therapeutic strategies targeting palmitoylation-modifying enzymes or their substrate proteins may enable precise regulation of inflammatory signals, presenting novel treatment approaches for multi-system inflammation-related disorders.

## 6 Potential clinical implication: focus on protein *S-*palmitoylation

The evidence presented above demonstrates that *S-*palmitoylation plays a crucial role in the physiological and pathological processes of numerous inflammation-related diseases, suggesting significant therapeutic potential. Current approaches to modify cellular *S-*palmitoylation levels primarily utilize two strategies: increasing *S-*palmitoylation or controlling depalmitoylation [[125], [126], [127]]. Table 1 [38,41,42,44,103,105,120,121,[128], [129], [130], [131], [132], [133], [134], [135], [136]] presents a summary of key molecules targeting palmitoylation-modifying enzymes.

**Table 1:** Modulators of palmitoylating and depalmitoylating enzymes.

Type	Compound	Regulatory target	Mechanism	Refs.
Broad-spectrum inhibitors	2-BP	Pan-inhibition of zDHHC	Inhibits zDHHC activity, thereby affecting the palmitoylation of inflammation-related targets such as NLRP3 and STAT3, and influencing inflammasome assembly/activation and T-cell differentiation	[41,105,128]
	CMA	Pan-inhibition of zDHHC	Suppresses zDHHC enzymatic activity	[129]
Selective inhibitors	PF-670462	zDHHC8	Promotes lysosomal degradation of zDHHC8	[134]
	ML348	APT1	Enhances GSDMD palmitoylation, which paradoxically increases cardiomyocyte pyroptosis in mice	[42,121]
	ML349	APT2	Inhibits APT2 activity, promotes MAVS palmitoylation and activation, thereby enhancing innate immune responses	[38,41]
	ABD957	ABHD17 A/B	Inhibits the enzymatic activity of ABHD17 protein through covalent modification of its active site, thereby suppressing the depalmitoylation of N-RAS, which in turn blocks N-RAS signal transduction and the growth of AML cells harboring NRAS mutations	[44]
	DC661	PPT1	DC661 directly binds to and inhibits the enzymatic activity of PPT1, thereby blocking its depalmitoylation function. This inhibition leads to enhanced palmitoylation of Gpx1, which in turn effectively regulates angiogenesis	[135]
	GNS561	PPT1	GNS561 induces hepatocellular carcinoma cell death by specifically inhibiting PPT1 to regulate lysosomal function	[136]
Substrate-specific agents	Disulfiram	NLRP3	Alters NLRP3 subcellular localization and inhibits inflammasome assembly/activation	[130]
		GSDMD	Blocks palmitoylation at GSDMD Cys192, limiting its ability to induce pyroptosis	[121]
	NU6300	GSDMD	Covalently binds to GSDMD Cys191, inhibits palmitoylation of full-length GSDMD and GSDMD-N domain, and disrupts GSDMD membrane localization and oligomerization	[131]
Substrate-specific agonists	Vaccarin	NLRP3	Promotes interaction between NLRP3 and zDHHC12, enhances NLRP3 palmitoylation, and induces autophagic degradation of NLRP3 inflammasomes	[120]
Peptide inhibitors	TIM-3 peptide	TIM-3	Blocks binding of zDHHC9 to TIM-3 Cys296, reversing T-cell exhaustion	[132]
	PD-PALM	PD-L1	Competitively inhibits zDHHC3 binding to PD-L1 Cys272, promoting PD-L1 ubiquitination and degradation	[103]
	CPPtat-S2	CD38	Competitively suppresses interaction between zDHHC9 and CD38, specifically inhibiting CD38 palmitoylation	[133]

2-BP: 2-bromopalmitate; zDHHC: zinc finger Asp-His-His-Cys motif-containing; NLRP3: nucleotide oligomerization domain (NOD)-like receptor thermal protein domain associated protein 3; STAT3: signal transducer and activator of transcription 3; CMA: *N*-cyanomethyl-*N*-myristamide; APT1: acyl protein thioesterase 1; GSDMD: gasdermin D; MAVS: mitochondrial antiviral-signaling protein; TIM-3: T-cell immunoglobulin and mucin-domain containing protein 3; PD-PALM: programmed death-ligand 1 palmitoylation motif; PD-L1: programmed death-ligand 1; CPPtat-S2: cell-penetrating peptide tat fused to S2 peptide; CD38: cluster of differentiation 38.

Specifically, 2-BP, the most commonly utilized broad-spectrum inhibitor of zDHHCs (PATs) [128], effectively inhibits zDHHC-mediated *S-*palmitoylation of NLRP3, thus reducing intestinal inflammation [105]. Additionally, 2-BP regulates STAT3 palmitoylation cycles through zDHHC7 activity inhibition, reducing palmitic acid (PA)-induced inflammation [41]. Other palmitoylation inhibitors include cerulenin and 2-fluoropalmitic acid (2-FPA), though their roles in inflammatory regulation and immune cell activation remain less extensively studied [137]. These observations indicate that 2-BP achieves anti-inflammatory effects through broad zDHHC activity inhibition. Similarly, *N*-cyanomethyl-*N*-myristamide (CMA), featuring a novel scaffold, functions as a broad-spectrum zDHHC inhibitor with potential advantages over 2-BP [129]. However, CMA's optimal concentration requirements vary by target, potentially limiting its applications. These limitations of current *S-*palmitoylation inhibitors have prompted investigation of alternative therapies, particularly targeting specific protein substrates. For instance, disulfiram, traditionally used for chronic alcoholism treatment, inhibits NLRP3 palmitoylation at Cys126, affecting its subcellular localization and suppressing inflammasome activation [130]. It also prevents GSDMD palmitoylation at Cys192, limiting pyroptosis induction [121]. Vaccarin enhances NLRP3 palmitoylation by facilitating its interaction with zDHHC12, thereby deactivating the NLRP3 inflammasome [120]. NU6300 forms covalent bonds with GSDMD at Cys191, inhibiting palmitoylation of both full-length GSDMD and its N-terminal fragment (GSDMD-N), disrupting membrane localization, oligomerization, and pyroptosis [131]. Researchers have developed specific peptides, including programmed death-ligand 1 palmitoylation motif (PD-PALM), TIM-3, cell-penetrating peptide tat fused to S2 peptide (CPPtat-S2), to decrease target protein palmitoylation [103,132,133], and Zhou et al. [134] identified PF-670462, a small-molecule inhibitor promoting zDHHC8 degradation. However, the application of these specific inhibitors in inflammation requires additional research.

Given the significant role of palmitoylation in disease, the impact of depalmitoylating enzyme modulators on inflammation warrants careful consideration. Researchers have developed specific inhibitors for depalmitoylases, including ML348 and ML349, which selectively inhibit APT1 and APT2, respectively [39,42,138]. ML349 enhances innate immune responses by promoting MAVS palmitoylation and activation through APT2 inhibition [38]. Furthermore, ML349 can increase STAT3 palmitoylation, thereby affecting STAT3 signaling and colon carcinogenesis [41]. Notably, ML348 enhances cardiomyocyte pyroptosis in mice by promoting GSDMD palmitoylation [121]. These findings suggest that modulating depalmitoylase activity may improve the outcomes of inflammation-related diseases. Additionally, researchers have developed alternative depalmitoylase inhibitors. For instance, DC661 inhibits the activity of PPT1, which in turn enhances the palmitoylation of GPX1 [135]. Similarly, GNS561 can specifically suppress PPT1 activity, thereby exerting substantial therapeutic potential in hepatocellular carcinoma [136]. Additionally, ABD957 inhibits the palmitoylation of N-Ras in acute myeloid leukemia (AML) cells by blocking the activity of ABHD17 [44].

These findings demonstrate the substantial clinical application potential of *S-*palmitoylation. Further investigation of its mechanism in inflammation-related diseases may reveal new opportunities for targeted inhibitor development.

## 7 Conclusion and future perspectives

Recent years have witnessed significant advances in understanding protein *S-*palmitoylation's regulatory mechanisms in inflammation-related diseases. Acting as a “molecular switch” in inflammatory pathways, *S-*palmitoylation dynamically regulates the TLR-NFκB immune signaling cascade, JAK-STAT3 inflammatory signal transduction, and the activation of the NLRP3 inflammasome and pyroptotic pore formation in the NLRP3-GSDMD axis. These mechanisms significantly influence immune cell differentiation, development, and biological functions, establishing *S-*palmitoylation as a promising therapeutic target for inflammation-related disorders. Current intervention strategies targeting *S-*palmitoylation include broad-spectrum inhibitors such as 2-BP targeting the zDHHC family, enzyme-specific inhibitors like PF-670462 selectively inhibiting zDHHC8, and ML349 and ML348 selectively inhibiting depalmitoylases APT. Peptide-based inhibitors such as PD-PALM, TIM-3, and CPPtat-S2 disrupt interactions between zDHHC enzymes and their substrates, while established drugs like Disulfiram and Vaccarin indirectly regulate NLRP3 palmitoylation. Research has progressed from broad-spectrum inhibitors to enzyme-substrate pair-specific interventions, expanding therapeutic approaches through gene editing and metabolic regulation.

While *S-*palmitoylation demonstrates significant therapeutic potential in inflammation-related diseases, several challenges remain. The primary limitation is the absence of high-resolution structural data and a comprehensive targeting map for specific zDHHC, which impedes a detailed understanding of *S-*palmitoylation's role in diseases. Additionally, developing specific zDHHC inhibitors presents challenges due to the high similarity among zDHHC family members' catalytic domains. The most effective and widely used *S-*palmitoylation inhibitor, 2-BP, inhibits most zDHHC proteins and may interact with other proteins, limiting its clinical application potential. Although *S-*palmitoylation presents promising opportunities for treating inflammatory diseases, achieving precise regulation to prevent excessive or insufficient inflammatory responses remains a significant challenge.

## CRediT authorship contribution statement

**Sunan Li:** Writing – review & editing, Writing – original draft, Validation, Conceptualization. **Xinyue Dou:** Writing – original draft, Validation. **Jiamei Sun:** Writing – review & editing, Writing – original draft, Visualization. **Yongyuan Xiao:** Visualization. **Tianyang Wang:** Writing – review & editing, Writing – original draft, Validation. **Qiyuan Shan:** Writing – review & editing. **Xin Han:** Writing – review & editing, Writing – original draft, Funding acquisition, Conceptualization. **Gang Cao:** Writing – review & editing, Supervision.

## Declaration of competing interest

The authors declare that they have no known competing financial interests or personal relationships that could have appeared to influence the work reported in this paper.
